# Micro- and Macroelemental Composition and Safety Evaluation of the Nutraceutical *Moringa oleifera* Leaves

**DOI:** 10.1155/2014/786979

**Published:** 2014-07-22

**Authors:** I. J. Asiedu-Gyekye, S. Frimpong-Manso, C. Awortwe, D. A. Antwi, A. K. Nyarko

**Affiliations:** ^1^Department of Pharmacology and Toxicology, College of Health Sciences, University of Ghana School of Pharmacy, P.O. Box LG 43, Legon, Ghana; ^2^Department of Pharmaceutical Chemistry, College of Health Sciences, University of Ghana School of Pharmacy, P.O. Box LG 43, Legon, Ghana; ^3^Division of Clinical Pharmacology, Faculty of Health Sciences, University of Stellenbosch, P.O. Box 19063, Cape Town, South Africa; ^4^Department of Physiology, College of Health Sciences, University of Ghana Medical School, P.O. Box 4236, Korle-Bu, Ghana

## Abstract

*Moringa oleifera* is a multipurpose plant used in Ghana and most parts of Africa. Its high mineral, protein, and vitamins content has enabled its use as a nutraceutical and panacea for various diseases. This study aimed at measuring the micro- and macroelements content of dried *Moringa oleifera* leaves using energy dispersive X-ray fluorescence spectroscopic (EDXRF) and assessing its toxicological effect in rats. Acute toxicity (5000 mg/kg) and a subacute toxicity studies of the leaf (40 mg/kg to 1000 mg/kg) extract were conducted in rats. Blood samples were assessed for biochemical and haematological parameters. Results showed significant levels of thirty-five (35) elements (14 macroelements and 21 microelements) in *M. oleifera* extract. There were no observed overt adverse reactions in the acute and subacute studies. Although there were observed elevations in liver enzymes ALT and ALP (*P* < 0.001) and lower creatinine levels in the extract treated groups, no adverse histopathological findings were found. *Moringa oleifera* dried leaf extract may, therefore, be reasonably safe for consumption. However, the consumption of *Moringa oleifera* leaves should not exceed a maximum of 70 grams per day to prevent cumulative toxicity of these essential elements over long periods.

## 1. Introduction

*Moringa oleifera* Lam. found in most parts of Ghana belongs to the monogenetic family Moringaceae (order Brassicales). It is a plant that has multipurpose, nonmedicinal, and medicinal uses. Its nonmedical uses include use of the seeds in wastewater treatment due to their coagulant properties [1, 2]. Its medicinal uses stem from the fact that the entire plant has high protein, vitamins, mineral, and carbohydrate content. It is, thus, of high nutritional value for both humans and livestock. Moringa leaves are rich in minerals such as iron, potassium, and calcium as well as vitamins, essential amino acids, and a number of glycosides [3, 4]. The seeds have high content (42%) of edible oil that also has medicinal uses.

Moringa is used for the management of various ailments, as a galactogogue in mothers of preterm infants [5, 6]. It is also used to manage heart diseases and eye problems as well as inflammations and dyspepsia [7, 8].

Pharmacological studies have shown that the extracts of the plant have antioxidant [9–11], anticarcinogenic [12], anti-inflammatory, antispasmodic and antidiuretic [13] properties. Others include antiulcer, antibacterial and antifungal properties [14]. Recent studies indicate that it also has antinociceptive [7] as well as wound healing ability [8]. Studies on the root bark have shown it to have analgesic, alexeteric, and antihelminthic properties. It has also been reported to alter blood lipid profiles [15]. Toxicity studies have shown that aqueous extract of moringa leaf extract has no significant adverse effects in rats, rabbits [15–18] or poultry [18]. However, there are significant differences in the safety and composition of various* moringa species* from different locations [19].

In Ghana and most parts of West Africa, powdered leaves of* M. oleifera* are marketed under different brand names and consumed daily as a nutraceutical, prophylaxis, or cure for various conditions. The prevailing socio-cultural/economic conditions and consequent difficulty in accessing healthcare services, especially by rural populations as well as the general perception that plant medicines are efficacious and free from side effects [19, 20] will increase the frequent and widespread spread use of moringa leaf powder. In view of the reported differences in moringa leaf products, which may impact adversely on its safety, the micro- and macroelemental content of dried leaves of* Moringa oleifera* were measured. In addition, the leaf extract was investigated for its toxicological effects in Sprague-Dawley rats.

## 2. Materials and Methods

### 2.1. Preparation of Extracts of* Moringa oleifera* Leaves for Analysis

In order to mimic the traditional method of extraction, 2.8 kg of sample were blended with boiled distilled water. The mixture, covered with water, was left to stand overnight in a water bath maintained at 60°C and the watery portion (infusion) was filtered off using 0.45 *μ*m millipore cellulose ester filters and freeze dried to obtain* M. oleifera* extract (MOE). Portions were homogenized and labelled as sample A for elemental analysis.

### 2.2. Preparation of* Moringa oleifera* Leaves for Analysis

Leaves of the* Moringa oleifera* species were collected from Accra, separated from other plant parts, washed and dried in the shade for five days at room temperature, ground into fine powder, sieved (212 *μ*m mesh size) to obtain very fine samples, and kept in separate containers. This was labelled sample B.

### 2.3. Pelleting of Samples for Analysis

The loose/powdery nature of samples (plant extracts A and leaf powder B) required that they were pelleted before analysis. Before pelleting, 4 g of each sample was weighed and 0.9 g of the binder Fluxana (H Elektronic BM-0002-1 (Licowax C micropowder PM-Hoechstwax)) was added and homogenized for 3 minutes. The mixture was pressed at 20 t or 2 minutes into pellets of 32 mm in diameter for the subsequent XRF measurements. Three separate pellets were prepared from each of samples A and B.

### 2.4. Energy Dispersive X-Ray (ED XRF) Measurements

Energy dispersive X-ray (ED XRF) was used for simultaneous analysis and measurement of the elemental content of the samples. The procedure, which used three-axial geometry, reduced background noise due to radiation polarization. The monochromatic radiations emitted from the X-ray tube were applied to excite the atoms of the sample.

### 2.5. Experimental Animals and Housing Conditions

Male Sprague-Dawley rats (150 g–180 g body weight) were purchased from the Centre for Scientific Research into Plant Medicine, Mampong. The rats were housed in plastic cages with stainless steel tops in the animal care facility of the University of Ghana Medical School and kept under standard 12 h light and 12 h dark schedule where room temperature, humidity, and ventilation were controlled during the acclimatization period of seven (7) days.

### 2.6. Acute and Subacute Toxicity Studies

The reconstituted powdered MOE mixture was prepared using distilled water as the vehicle and administered as a single dose (5,000 mg/kg) by oral gavage for the acute toxicity studies. After the administration, animals were observed every hour for the first 6 hours then daily for the next 13 days.

In the subacute studies, five groups (1–5) of male Sprague-Dawley Rats (eight weeks old, seven animals/group) of mean weight 150 g were prepared. Animals in each of the four groups were administered MOE extract over a dose range of 0 mg/kg to 1000/kg by oral gavage daily for 14 days. Animals in the fifth group received the vehicle (distilled water)and served as controls.

For the subacute studies, after the14 days of MOE administration of MOE, each animal was observed every hour for six hours daily for the next 3 days and subsequently every day for 10 days. Animals were fed* ad libitum* with standard chow diet (AIN-93G formulation obtained from GAFCO-Ghana).

#### 2.6.1. Observed Clinical Signs of Toxidromes

Body weights were observed daily before and after administration. In addition, animals were observed daily for clinical signs of excitability, twitching, salivation, morbidity, miosis, mydriasis, rising fur, sluggish movement, draping, tremors, and so forth.

### 2.7. Laboratory Examinations

#### 2.7.1. Blood Samples

Under chloroform anaesthesia, blood was obtained via cardiac puncture and the animals euthanized by exsanguinations. Samples of the blood collected were aliquoted into EDTA-2K tubes and plain tubes, respectively. The EDTA blood was immediately analysed for haematological parameters using the SYSMEX Haematology Autoanalyser [Kobe, Japan] while serum prepared from blood in the plain tubes was used for biochemical examinations.

### 2.8. Necropsy and Histopathological Studies

Gross pathological investigations were conducted on each animal following exsanguination. The heart, kidney, liver, and gastrointestinal tract were prepared for histopathological examinations. All experimental procedures and assays were conducted in accordance with the international guidelines for evaluating the safety of herbal medicines [21–23].

### 2.9. Statistical Analysis

Results are presented as means ± SEM and analysis for statistical differences was done using one-way ANOVA followed by* Bonferroni post-hoc test*. *P* values less than 0.05 were considered statistically significant.

## 3. Results

The ED-XRF analyses led to the detection of a total of thirty-five (35) elements comprising eleven (11) major elements and twenty-four (24) minor elements. The major elements detected included Na, Mg, Al, Si, P, S, Cl, K, Ca, Mn, and Fe and other heavy metals. Tables 1 and 2 show details of the elements detected and their concentrations.

### 3.1. Clinical Symptoms

In the acute toxicity studies as well as the subacute studies at all the dose levels, grooming, repetitive circling with arched-back posture were observed in all the rats except those in the control group. Rats given high doses of MOE (1000 mg/kg and 5000 mg/kg) showed more excitability, twitching, and salivation. Necropsy revealed no abnormalities.

### 3.2. Haematology

These results of haematological investigations are presented in Table 3 (acute toxicity) and Table 4 (subacute toxicity studies). In the acute toxicity studies, WBC increased significantly (by 52.5%). MCV, on the other hand, decreased by 10%. Similarly, platelets levels dropped significantly (62.5%) following treatment with MOE.

In the subacute studies, WBC increased by 73.75% and 67% (*P* < 0.0001) at dose levels of 40 mg/kg and 80 mg/kg, respectively. MCV dropped significantly at the 200 mg/kg and 1000 mg/kg dose levels. Similarly, platelets levels rose significantly over baseline level at the 80 mg/kg dose level. However, at 1000 mg/kg, they dropped significantly.

### 3.3. Biochemical Analysis

**Figure 1:**
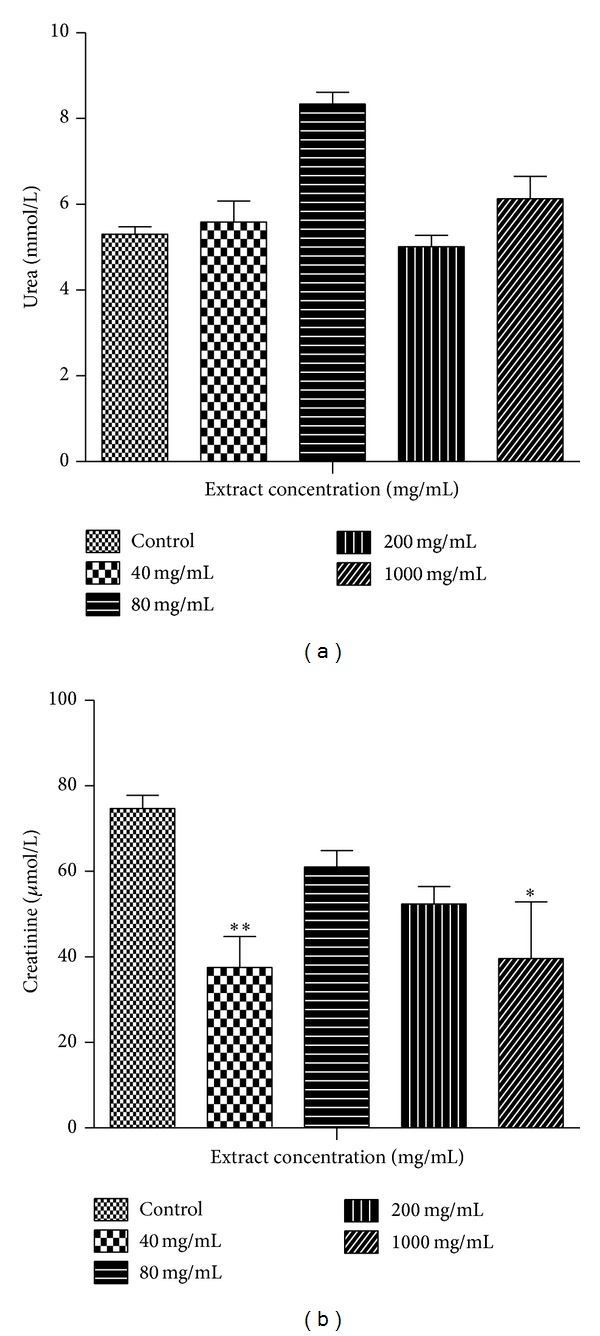
Renal function test during a 14-day administration of MOE in male SDRs. Values are expressed as means ± SEM (*n* = 7). Values of *P* < 0.05 were considered as statistically significant. *P < 0.05, **P < 0.01, and ***P < 0.0001 when control was compared with MOE.

Figure 1 shows results obtained from the biochemical parameters used as markers for renal function. The figure shows that blood urea levels were elevated at dose levels of 80 mg/kg and 1000 mg/kg. Creatinine levels, however, significantly decreased at all dose levels compared to the controls.

**Figure 2:**
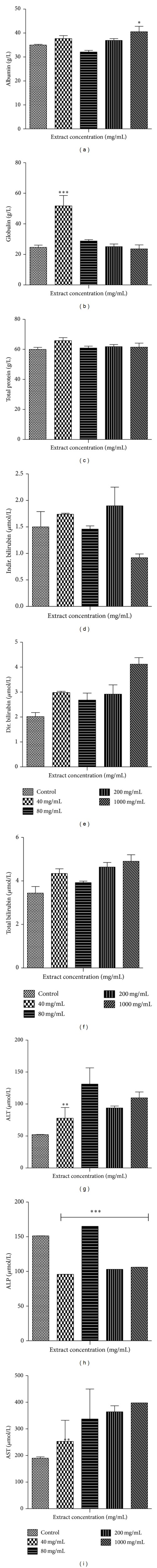
Liver function tests of a 14-day administration of MOE in male SDRs. Values are expressed as means ± SEM (*n* = 7). Values of *P* < 0.05 were considered as statistically significant. *P < 0.05, **P < 0.01, and ***P < 0.0001 when control was compared with MOE.

**Figure 3:**
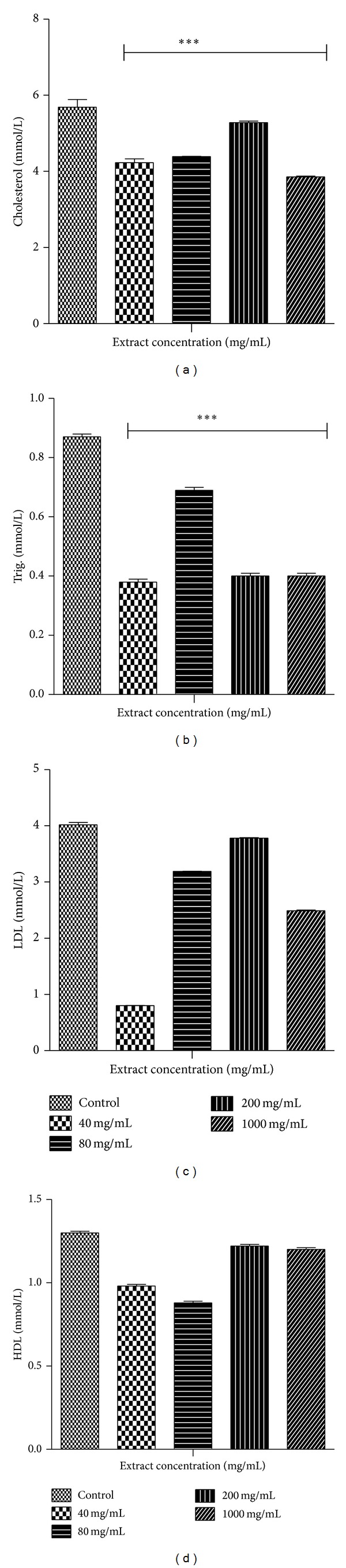
Lipid profile of a 14-day administration of MOE in male SDRs. Values are expressed as means ± SEM (*n* = 7). Values of *P* < 0.05 were considered as statistically significant. *P < 0.05, **P < 0.01, and ***P < 0.0001 when control was compared with MOE.

Figures 2 and 3 show results for the biochemical test for liver function and lipid profiles, respectively. There were increases in the levels of liver enzymes during MOE administration in a non-dose dependent manner. AST levels, however, reduced at all dose levels except the 80 mg/kg group. There were decreases in the lipid parameters during MOE administration. For the lipid profiles, MOE administration generally led to a decrease in total cholesterol and triglycerides as well as LDL and, to some extent, HDL cholesterol.

### 3.4. Histopathology

Photomicrographs of tissues prepared from control and animals treated with MOE at different dose levels are presented in Figures 5–8. No observable cardiomyopathy was noted in the heart (Figure 5).  Figure 6 showed no observable ulceration. The epithelial cells of the stomach are intact.  Figure 7 also showed no observable histological lesions in the glomerulus and the tubules. There were no observable histological lesions in the sinusoids and central vein of the liver (Figure 8).

## 4. Discussion

Normally, herbal preparations are considered relatively safe and devoid of numerous adverse effects probably because they are considered to be “natural.” This study attempted to analyse the elemental content of* Moringa oleifera* and to ascertain if a 14-day dosing could have any observable adverse effects. This is in view of the widespread use of moringa leaf powder as a food supplement and treatment for various disease conditions.

**Figure 4:**
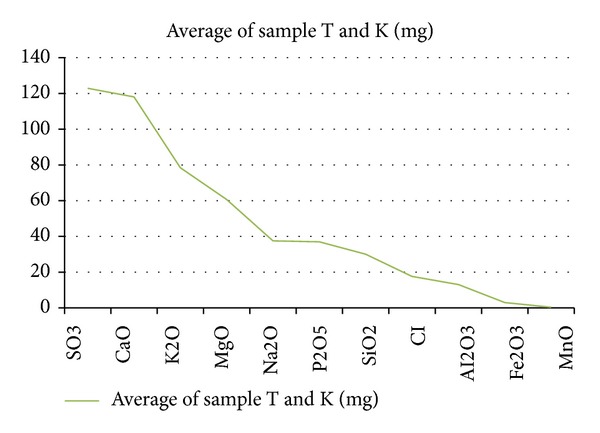
Chart showing the decreasing order of concentration of elements in the leaves of* Moringa oleifera*.

X-ray fluorescence (XRF) is a fast, accurate, and nondestructive analytical technique used for the elemental and chemical analysis of powdered, solid, and liquid samples. Analyses of the samples produced a total of 35 elements (14 macroelements and 21 microelements) in* M. oleifera*. The concentrations of macroelements in the powdered leaf samples are shown in Figure 4 and included S, Ca, K, Mg, Na, P, Si, Cl, Al, Fe, and Mn. The minor elements in the decreasing order were V, Ba, Cr, Y, Ba, Zn, Rb, Ce, La, Cu, Cs, Sn, Hf, Co, Ni, Nb, Ta, U, Mo, Pb, Bi, Ga, Th, As, and Zr. All concentrations were within the recommended daily allowance (RDA) limits. RDA, the average daily dietary intake level, is expected to be sufficient to meet the nutrient requirements of all healthy individuals [24–26]. These results, therefore, would suggest that consumption of moringa leaf powder can provide users with some of the essential minerals that the human body requires for optimum function.

Blood parameter analysis is relevant to risk evaluation as the haematological system has a higher predictive value for toxicity in humans (91%) [27].* Moringa oleifera* extract administration was accompanied by a reduction in the haematopoietic system (Tables 3 and 4) at a dosage of 40 mg/kg which is evidenced by the levels of RBC, HCT, HGB, WBC, and lymphocytes. The slightly reduced HGB, HCT, RBC, and total protein in the rats suggest potential interference with the haematopoietic system that would require further investigations. The high RBC could also be due to hypoxic conditions that might have resulted due to the high doses of MOE administered. The absence of a major significant change in haematological parameters is consistent with observations by Awodele et al. [16].

The statistically significant increase in lymphocytes at MOE doses of 40–80 mg/kg and the 5,000 mg/kg BW in the animals might suggest the presence of infection/stress or a potential immune boosting effect of MOE at the stated dose levels. Since the animals were specific pathogen-free and were kept in a barrier system, the observed effects are likely to be due to potential immune boosting effects of the extract [13, 14, 28]. This needs further investigation in a subchronic or chronic study.

AST, ALT, and ALP are major markers of liver function. Toxic injury to the liver leads to elevation in levels of all these liver enzymes. Thus, the observed rise in ALT and ALP following administration of MOE (Tables 5 and 6) would suggest a potential adverse effect of MOE on the liver. Since ALT is localized primarily in the cytosol of hepatocytes, it is a more sensitive marker of hepatocellular damage than AST and ALP [27, 28]. Withinlimits, these can provide a quantitative assessment of the degree of damage sustained by the liver. Histopathological examinations, however, did not reveal anyhistological lesionsin the sinusoids or central vein (Figure 8).

The copper component in the extract (Table 2) which is a component of a number of enzymes that are involved in reducing molecular oxygen, metabolizing substances such as histamine, serotonin, epinephrine, norepinephrine, and dopamine could pose a threat during abnormal consumption of the extract. Copper deficiency, although rare, results in hypochromic anaemia [26]; possible side-effects may include liver damage and Wilson's syndrome.

Direct, indirect, and total bilirubin reflects the liver's ability to take up, process, and secrete bilirubin into bile and can also be considered as a true test of liver function [27, 28]. These were all high at the different doses, especially at the 5,000 mg/kg single oral high dose compared to the controls (Tables 5 and 6). However, total protein, globulin, and albumin remained relatively unchanged or slightly increased in all the groups in comparison with the controls. These were not entirely dose dependent and significant but require further monitoring in subchronic studies.

It must also be noted that gross pathological examination of the treated animals did not reveal any abnormalities such as presence of lesions or changes in colour of internal organs and relative organ weights as compared to the controls.

The kidneys are concerned with the elimination of drugs from the body and are likely to be affected during such toxicity studies at high doses. In the treated male rats, creatinine levels were reduced compared to the controls (*P* < 0.05) while urea levels were inconsistent among the groups. This suggests that MOE did not adversely affect the integrity of the renal system [29]. Also, histopathological examinations did not reveal any observable histological lesions in the glomeruli and the tubules (Figure 7). However, the reduction in creatinine levels may be deemed to be positive effect of MOE on the renal system, which could be exploited therapeutically. These findings are consistent with those reported by Isitua and Ibeh, 2013 [17].

**Figure 5:**
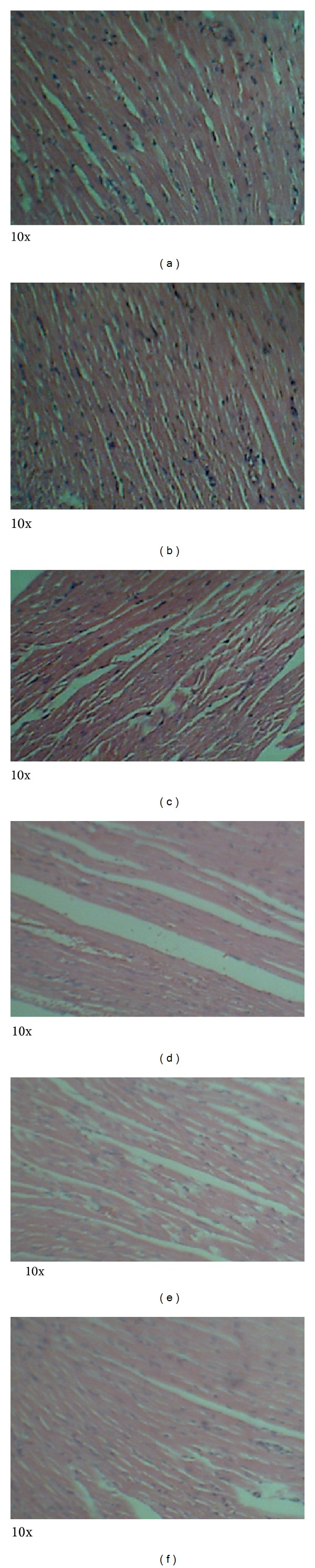
Photomicrographs of the heart from male Sprague-Dawley rats. No observable cardiomyopathy. Keys: (a): control, (b): 5000 mg/kg bwt, (c): 1000 mg/kg bwt, (d): 200 mg/kg bwt, (e): 80 mg/kg bwt, and (f): 40 mg/kg bwt.

**Figure 6:**
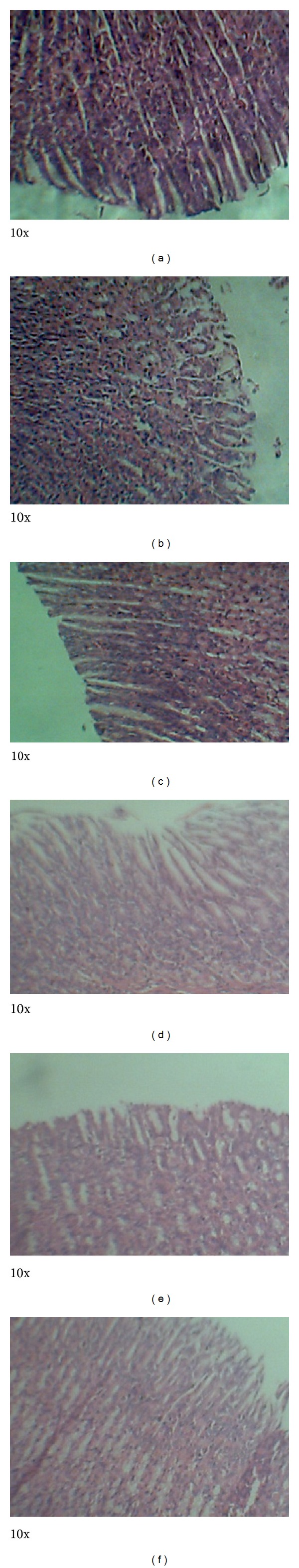
Photomicrographs of the stomach from male Sprague-Dawley rats. No observable ulceration. The epithelial cells are intact. Keys: (a): control, (b): 5000 mg/kg bwt, (c): 1000 mg/kg bwt, (d): 200 mg/kg bwt, (e): 80 mg/kg bwt, and (f): 40 mg/kg bwt.

**Figure 7:**
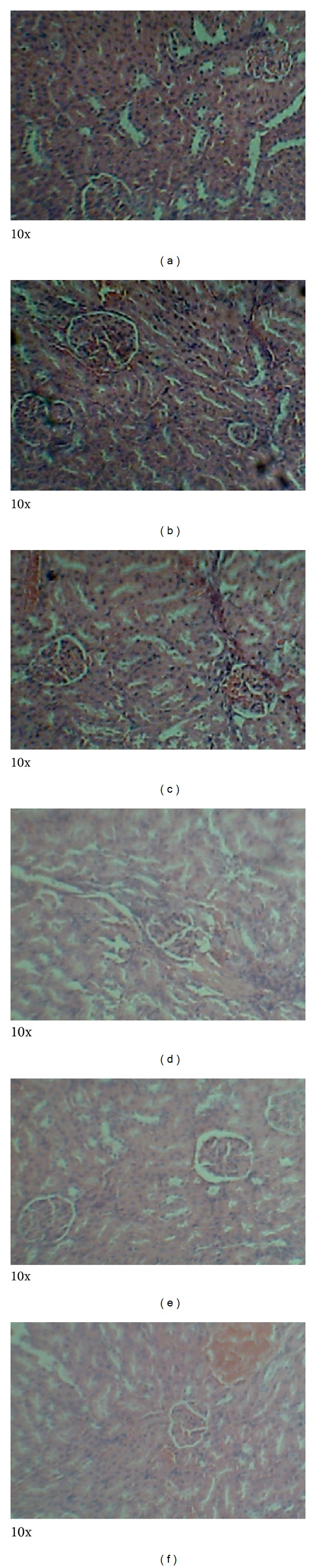
Photomicrographs of the kidney from male Sprague-Dawley rats. There were no observable histological lesions in the glomerulus and the tubules. Keys: (a): control, (b): 5000 mg/kg bwt, (c): 1000 mg/kg bwt, (d): 200 mg/kg bwt, (e): 80 mg/kg bwt, and (f): 40 mg/kg bwt.

**Figure 8:**
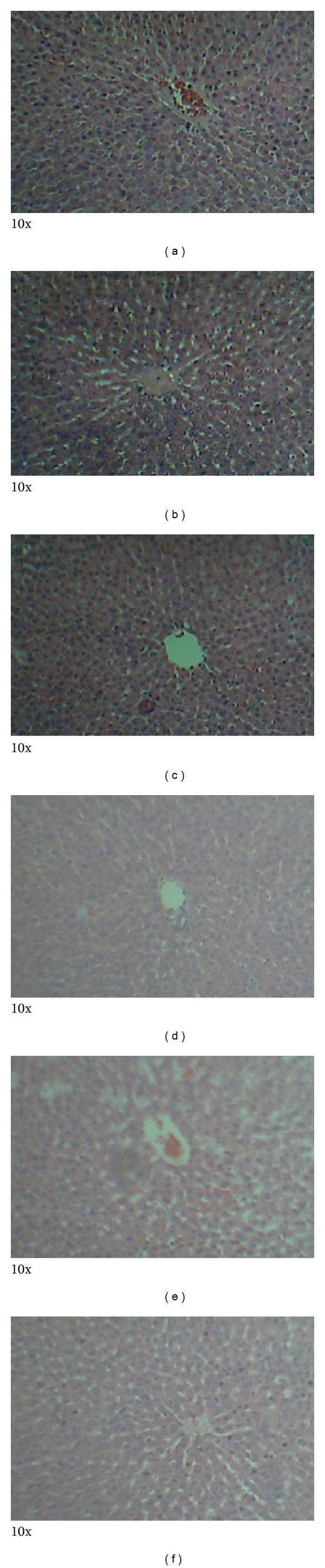
Photomicrographs of the liver from male Sprague-Dawley rats. There were no observable histological lesions in the sinusoids and central vein. Keys: (a): control, (b): 5000 mg/kg bwt, (c): 1000 mg/kg bwt, (d): 200 mg/kg bwt, (e): 80 mg/kg bwt, and (f): 40 mg/kg bwt.

Ingestion of chemicals substances including those of plant origin in excess and especially over long periods may adversely affect major organs like the heart, kidney, liver, and even the gastrointestinal system. Thus, the evaluation of histopathological changes in organs remains a cornerstone in assessing the safety of medicines and other substances [30, 31]. Histopathological examinations of the heart and stomach did not reveal any observable cardiomyopathies (Figure 5) and ulcerations of the epithelial cells in the male rats (Figure 6). These findings are consistent with other studies [32]. It might be expected that at high doses of MOE may result in accumulation of MOE-derived iron. This mineral, though an important component of haemoglobin and other proteins and enzymes, may cause gastrointestinal distress, hemochromatosis, and so forth [22, 23]. The absence of these effects may be explained by the fact that the various elements were within normal limits [16].

It is important to note that the effects of chemicals produced in laboratory animals when properly conducted provide a useful indication of safety in humans [33]. Thus, from the above results, it would be expedient to conduct subchronic or chronic toxicity studies with regard to the hematopoietic, renal, hepatic, and reproductive changes because MOE is used as a food supplement and is used over a long period of time by consumers. Moreover, the contamination of moringa leaves by heavy metals like lead (Pb) and arsenic (As) may pose a threat to humans because they are not biodegradable. With reference to the RDA limits and other studies conducted regarding the presence of heavy metals in the leaves of MOE [34, 35], it is imperative that prevention of any possible cumulative toxicity of some of the elements is most desirable during prolonged use of MOE. There is, thus, the need for toxicological studies to be conducted on moringa from different locations in order to give an indication of their comparative safety.

It must, however, be stated that, because plants may absorb elements from the soil and environment, some of which may be toxic to humans, plant nutrition, climate, and soil conditions and locations could also determine the elemental contents in the leaves [19, 34, 35].

## 5. Conclusion

This study has provided some evidence that* Moringa oleifera* that was collected in Accra, Ghana, in West Africa is reasonably safe for consumption taking into account the elemental composition when administered to rats. The acute studies showed that the median lethal dose (LD_50_) could be greater than 5000 mg/kg as all the animals survived at his dose level. It is also important to monitor the concentration of its elemental composition as a possible reason of serious health alterations.

## 6. Recommendation

Based on the levels of these minerals and the permissible amount in the human body, it is recommended that the consumption of* Moringa oleifera* leaves be limited to a maximum of 70 grams per day in order to prevent excessive consumption and subsequent accumulation of some of these essential elements. At 70 grams per day, most of these elements in the leaves could be found in high quantities approaching the RDA limit.

## Figures and Tables

**Table 1:** Concentrations of major oxides per total mass of powdered leaf in milligrams.

Major oxides	Level in sample (mg)
Na_2_O	37.5
MgO	60.6
Al_2_O_3_	13.0
SiO_2_	30.0
P_2_O_5_	37.0
SO_3_	122.8
Cl	17.6
K_2_O	78.4
CaO	118.0
MnO	0.4
Fe_2_O_3_	3.0

**Table 2:** Concentrations of minor elements per total mass of powdered leaf in milligrams.

Minor elements	Level in sample (mg)
V	2.400
Cr	0.578
Co	0.012
Ni	0.0148
Cu	0.0318
Zn	0.1156
Ga	0.0032
As	0.0022
Rb	0.0758
Y	0.3542
Zr	0.0024
Nb	0.0116
Mo	0.0056
Sn	0.0576
Cs	0.0242
Ba	0.8900
La	0.0490
Ce	0.0694
Hf	0.0154
Ta	0.0114
Pb	0.0044
Th	0.0032

**Table 3:** Haematological analysis of a 5,000 mg/kg single dose administration of MOE in male SDRs. Values are expressed as mean ± SEM (*n* = 7). Values of *P* < 0.05 were considered as statistically significant. *P < 0.05, **P < 0.01, and ***P < 0.0001 when control was compared with MOE.

Parameters	Groups	
	Control males	5000 mg/kg males
WBC (10^3^ *µ*L)	4.8 ± 0.32	7.32 ± 0.04***
RBC (10^6^ *µ*L)	8.3 ± 0.40	8.84 ± 0.04
HGB (g/dL)	15.1 ± 0.59	14.82 ± 0.04
HCT (%)	53.6 ± 2.31	52 ± 0.32
MCV (fL)	65 ± 0.38	58.2 ± 0.07***
MCH (pg)	18.3 ± 0.16	16.64 ± 0.04
MCHC (g/dL)	28.17 ± 0.16	28.6 ± 0.04
PLT (10^3^ *µ*L)	300.3 ± 44.44	112.8 ± 0.37**
LYM%	84.33 ± 1.39	83.14 ± 0.05
LYM number	4.1 ± 0.28	6.1 ± 0.04

**Table 4:** Haematological Analysis of a 14-day administration of MOE in male SDRs. Values are expressed as mean ± SEM (*n* = 7). Values of *P* < 0.05 were considered as statistically significant. *P < 0.05, **P < 0.01, and ***P < 0.0001 when control was compared with MOE.

Parameters	Groups					
	Control males	40 mg/kg males	80 mg/kg males	200 mg/kg males	1000 mg/kg males	*P* value
WBC (10^3^ *µ*L)	4.8 ± 0.32	8.34 ± 0.02***	7.2 ± 0.03***	4.2 ± 0.32	4.6 ± 0.14	0.0001
RBC (10^6^ *µ*L)	8.3 ± 0.40	6.06 ± 0.08***	6.55 ± 0.49*	7.71 ± 0.04	7.64 ± 0.19	0.0001
HGB (g/dL)	15.1 ± 0.59	10.7 ± 0.04	13.75 ± 0.08	13.74 ± 0.04	12.8 ± 0.66	0.934
HCT (%)	53.6 ± 2.31	39 ± 0.49	41.2 ± 3.25	46.22 ± 0.75	46.8 ± 1.71	0.0451
MCV (fL)	65 ± 0.38	65.14 ± 0.22	62.78 ± 0.73*	61.27 ± 0.16***	61.1 ± 0.66***	0.0001
MCH (pg)	18.3 ± 0.16	18.66 ± 0.02	18.5 ± 0.09	17.74 ± 0.07	16.73 ± 0.61	0.9705
MCHC (g/dL)	28.17 ± 0.16	28.36 ± 0.02	29.25 ± 0.17	28.6 ± 0.24	27.32 ± 0.93	0.9846
PLT (10^3^ *µ*L)	300.3 ± 44.44	377 ± 20.87	577.2 ± 45.78***	311.2 ± 3.96	137 ± 13.09**	0.0001
LYM%	84.33 ± 1.39	75.8 ± 0.04	60.02 ± 6.32*	82.42 ± 0.59	84.42 ± 0.97	0.0098
LYM number	4.1 ± 0.28	6.34 ± 0.05	8.9 ± 0.98	3.36 ± 0.24	3.78 ± 0.04	0.9535

**Table 5:** 

Parameters	Groups	
	Control males	5000 mg/kg males
Urea	5.3 ± 0.18	5.83 ± 0.8
Creatinine	74.7 ± 3.07	27.14 ± 0.87***
T. protein	59.96 ± 1.44	67.5 ± 2.90
Albumin	35 ± 0.31	36.08 ± 0.99
Globulin	24.66 ± 1.46	31.16 ± 2.3
Dir. Bil.	2.02 ± 0.16	5.42 ± 1.00***
Indirct. Bil.	1.5 ± 0.29	2.72 ± 0.59
T. Bil.	3.44 ± 0.29	6.16 ± 1.14*
ALT	52.28 ± 0.29	113.5 ± 7.19
AST	190.1 ± 4.96	224.4 ± 53.89
ALP	151.2 ± 0.196	276.9 ± 0.045***
Cholesterol	5.69 ± 0.2	4.18 ± 0.01***
Trig.	0.87 ± 0.01	0.4 ± 0.003***
LDL	4.02 ± 0.04	3.11 ± 0.004***
HDL	1.30 ± 0.01	0.88 ± 0.01

These are statistical significance: *P < 0.05; **P < 0.01; ***P < 0.0001.

**Table 6:** 

Parameters	Groups					
	Control males	1000 mg/kg males	200 mg/kg males	80 mg/kg males	40 mg/kg males	*P* value
Urea	5.3 ± 0.18	6.13 ± 0.52	5.01 ± 0.27	8.34 ± 0.27	5.59 ± 0.49	0.2143
Creatinine	74.7 ± 3.07	39.66 ± 13.23∗	52.4 ± 4.03	61.04 ± 3.84	37.52 ± 7.27∗∗	0.0005
T. protein	59.96 ± 1.44	61.52 ± 2.68	61.82 ± 1.38	60.92 ± 1.26	65.84 ± 1.98	0.0913
Albumin	35 ± 0.31	40.56 ± 2.24∗	36.92 ± 0.75	32.12 ± 0.66	37.7 ± 1.23	0.0016
Globulin	24.66 ± 1.46	23.64 ± 2.60	25.1 ± 1.71	28.80 ± 0.91	51.74 ± 6.83∗∗∗	0.0001
Dir. bil.	2.02 ± 0.16	4.12 ± 0.26	2.92 ± 0.37	2.68 ± 0.28	2.98 ± 0.05	0.0004
Indirdt. bil.	1.5 ± 0.29	0.92 ± 0.07	1.9 ± 0.35	1.46 ± 0.06	1.74 ± 0.02	0.0120
T. bil.	3.44 ± 0.3	4.9 ± 0.30	4.64 ± 0.21	3.92 ± 0.07	4.34 ± 0.22	0.0188
ALT	52.28 ± 0.29	109.8 ± 9.06	93.92 ± 2.93	131.3 ± 25.29∗∗	77.98 ± 16.67	0.0049
AST	190.1 ± 4.96	397.5 ± 0.04	364.5 ± 22.21	337.3 ± 112.4	253.3 ± 78.91	0.1928
ALP	151.2 ± 0.196	106.0 ± 0.049∗∗∗	103.1 ± 0.04∗∗∗	165.1 ± 0.037∗∗∗	95.84 ± 0.051∗∗∗	0.0001
Cholesterol	5.69 ± 0.2	3.86 ± 0.02∗∗∗	5.28 ± 0.04∗∗∗	4.39 ± 0.005∗∗∗	4.23 ± 0.10∗∗∗	0.0001
Trig.	0.87 ± 0.01	0.4 ± 0.01∗∗∗	0.4 ± 0.01∗∗∗	0.69 ± 0.01∗∗∗	0.38 ± 0.01∗∗∗	0.0001
LDL	4.02 ± 0.04	2.49 ± 0.01	3.78 ± 0.01	3.19 ± 0.004	0.8 ± 0.005	0.0001
HDL	1.30 ± 0.01	1.20 ± 0.01	1.22 ± 0.01	0.88 ± 0.01	0.98 ± 0.01	0.0001

These are statistical significance: *P < 0.05; **P < 0.01; ***P < 0.0001.
